# Declining Efficacy of Artemisinin Combination Therapy Against *P. Falciparum* Malaria on the Thai–Myanmar Border (2003–2013): The Role of Parasite Genetic Factors

**DOI:** 10.1093/cid/ciw388

**Published:** 2016-06-16

**Authors:** Aung Pyae Phyo, Elizabeth A. Ashley, Tim J. C. Anderson, Zbynek Bozdech, Verena I. Carrara, Kanlaya Sriprawat, Shalini Nair, Marina McDew White, Jerzy Dziekan, Clare Ling, Stephane Proux, Kamonchanok Konghahong, Atthanee Jeeyapant, Charles J. Woodrow, Mallika Imwong, Rose McGready, Khin Maung Lwin, Nicholas P. J. Day, Nicholas J. White, Francois Nosten

**Affiliations:** 1 Shoklo Malaria Research Unit, Mahidol-Oxford Tropical Medicine Research Unit, Faculty of Tropical Medicine, Mahidol University, Mae Sot, Thailand; 2 Centre for Tropical Medicine and Global Health, Nuffield Department of Medicine, University of Oxford, United Kingdom; 3 Mahidol Oxford Research Unit, Faculty of Tropical Medicine, Mahidol University, Bangkok, Thailand; 4 Department of Genetics, Texas Biomedical Research Institute, San Antonio; and; 5 Division of Molecular Genetics & Cell Biology, School of Biological Sciences, Nanyang Technological University, Singapore

**Keywords:** *Plasmodium falciparum* malaria, mefloquine–artesunate, *Pfmdr1*, *K13* mutation, artemisinin resistance

## Abstract

***Background.*** Deployment of mefloquine–artesunate (MAS3) on the Thailand–Myanmar border has led to a sustained reduction in falciparum malaria, although antimalarial efficacy has declined substantially in recent years. The role of *Plasmodium falciparum K13* mutations (a marker of artemisinin resistance) in reducing treatment efficacy remains controversial.

***Methods.*** Between 2003 and 2013, we studied the efficacy of MAS3 in 1005 patients with uncomplicated *P. falciparum* malaria in relation to molecular markers of resistance.

***Results.*** Polymerase chain reaction (PCR)–adjusted cure rates declined from 100% in 2003 to 81.1% in 2013 as the proportions of isolates with multiple *Pfmdr1* copies doubled from 32.4% to 64.7% and those with *K13* mutations increased from 6.7% to 83.4%. *K13* mutations conferring moderate artemisinin resistance (notably E252Q) predominated initially but were later overtaken by propeller mutations associated with slower parasite clearance (notably C580Y). Those infected with both multiple *Pfmdr1* copy number and a *K13* propeller mutation were 14 times more likely to fail treatment. The PCR-adjusted cure rate was 57.8% (95% confidence interval [CI], 45.4, 68.3) compared with 97.8% (95% CI, 93.3, 99.3) in patients with *K13* wild type and *Pfmdr1* single copy*. K13* propeller mutation alone was a strong risk factor for recrudescence (*P* = .009). The combined population attributable fraction of recrudescence associated with *K13* mutation and *Pfmdr1* amplification was 82%.

***Conclusions.*** The increasing prevalence of *K13* mutations was the decisive factor for the recent and rapid decline in efficacy of artemisinin-based combination (MAS3) on the Thailand–Myanmar border.

## Abstract (precis)

The pivotal factor leading to the declining efficacy of the artemisinin-based combination on the Thailand–Myanmar border (mefloquine–artesunate) to a clinically unacceptable level is the increasing local prevalence of *K13* mutations superimposed onto a long-standing background of *Pfmdr1* amplification.

The Thailand–Myanmar border is endemic for malaria with high-grade antimalarial drug resistance [1, 2]. After the failures of chloroquine and sulfadoxine-pyrimethamine, mefloquine was introduced as the treatment of uncomplicated *Plasmodium falciparum* malaria in 1985. However, resistance developed rapidly [2, 3], mediated by amplification of the multidrug-resistance gene 1 (*Pfmdr1*) [4]. In 1992 the cure rate of high-dose mefloquine monotherapy had fallen to 50% [5]. The first artemisinin-based combination treatment (ACT), mefloquine plus artesunate (MAS3), was deployed in 1994 in camps for displaced people [2]. The new treatment was highly efficacious and accompanied by a large and sustained reduction in *P. falciparum* incidence [6, 7] despite continued presence of effective malaria vectors. A similar impact was documented in the population of migrant workers living along the border [8]. The diminishing proportion of infections carrying multiple parasite clones also suggested reduced transmission [9].

Resistance to artemisinin derivatives, characterized by delayed parasite clearance in artesunate-treated patients, was first recognized in western Cambodia in 2007 [10, 11] and later on the Thailand–Myanmar border where it was found to be heritable [12] and associated with a selective sweep on chromosome 13 of the *P. falciparum* genome [13] due to mutations in the propeller region of the Kelch gene (*K13*) [14, 15]. However, the link between *K13* polymorphism and treatment failure was not clearly established, and some authors have contested whether use of the term “artemisinin resistance” is justified [16–18]. This uncertainty may have contributed to the failure to contain artemisinin resistance in the greater Mekong area.

Two recent studies have provided some evidence that *K13* mutations are involved in combination with other mutations in the declining efficacy of 3-day dihydroartemisinin (DHA)–piperaquine in Cambodia [19]. An observational cohort study reported that treatment failure rates were higher in patients infected with parasites carrying the *K13* C580Y mutation compared with the R539T mutation, but there were only 4 wild-type infections in that study. Two additional mutations (MAL13:1718319 and MAL10:688956), associated with parasite clearance rate in a genome wide association (GWAS) study [20], were in strong linkage disequilibrium with C580Y. The authors concluded that this triple mutant genotype might have contributed to the failures observed. A second study of 241 patients showed falciparum malaria recrudescence was associated with increased piperaquine in vitro 50% inhibitory concentrations and the presence of *K13* mutations at 3 Cambodian sites [21] where isolates with multiple copies of *Pfmdr1* are rare [21].

In contrast to Cambodia, *P. falciparum* malaria parasites along the Thailand–Myanmar border frequently have multiple copies of *Pfmdr1* [4, 22]. The MAS3 combination has been deployed successfully as a first-line regimen for more than 15 years, but failure rates in recent years have risen. To determine the factors contributing to declining ACT efficacy, we studied 1005 falciparum malaria patients treated with MAS3 between 2003 and 2013.

## METHODS

### Patient Recruitment

The study was designed for the longitudinal monitoring of MAS3 efficacy in prospectively enrolled patients presenting to the clinics of the Shoklo Malaria Research Unit [23] with uncomplicated *P. falciparum* malaria, excluding pregnant women, patients with severe malaria [24] or >4% infected red blood cells, and those who had been treated with mefloquine in the previous 60 days. All patient treatments were supervised. Oral artesunate (Guilin Pharmaceutical Co., People's Republic of China), was given at a dose of 4 mg/kg/day for 3 days. Mefloquine (Roche, Switzerland, or CIPLA, India) was given either as split doses of 15 mg/kg and 10 mg/kg or 8 mg/kg once a day for 3 days [25]. On the first treatment day (day 0) a full clinical examination was performed; parasitemia and hematocrit were measured from capillary blood. Blood smears were microscopically examined daily until negative. Patients were seen weekly for 6 weeks. At each visit symptoms were recorded and a capillary blood sample was obtained for malaria smear and hematocrit. Dried blood spots for parasite genotyping were collected on grade 3 MM Whatman paper (Whatman, United Kingdom) at the first visit and in case of recurrence. Recurrence was defined by the occurrence of parasitemia during follow-up due either to a recrudescence or a reinfection. In 2013 single-dose primaquine as a gametocytocide (0.25 mg/kg) was added routinely on the first day of treatment.

### Parasite Genotyping

Parasite DNA was extracted and genotyped at 3 polymorphic loci (MSP1, MSP2, and GLURP) to distinguish recrudescence from reinfection [26]. We determined *Pfmdr1* copy number using quantitative polymerase chain reaction (PCR) and *K13* polymorphisms by direct sequencing of PCR products (see Supplementary Methods). *Pfmdr1* and *K13* sequencing were done retrospectively after patient recruitment was completed.

### Statistical Analyses

Data were analyzed using Stata 14 (StataCorp, College Station, Texas). Normally distributed data were compared using Student *t* test and nonnormally distributed data were compared using the Mann–Whitney rank sum test. Categorical variables were assessed using χ^2^ tests. Analysis of variance was used to compare 3 or more groups. We used logistic regression to examine the association between each potential risk factor and used outcome and multiple logistic regression to analyze resulting risk factors. Survival time data were assessed using Cox regression. The population attributable fraction (PAF) for falciparum malaria recrudescence was calculated for *K13* mutations and *Pfmdr1* amplification individually and then for the marker combination using the formula 1–(1–PAF*_K13_*) × (1–PAF*_Pfmdr1_*). We censored patients with indeterminate genotypes, new infections with *P. falciparum*, or those lost to follow-up.

### Ethical Approval

The Oxford Tropical Research Ethics Committee (OXTREC 562-15) and the faculty of Tropical Medicine, Mahidol University (MUTM 2015-019-01) gave ethical approval for the study.

## RESULTS

### Patients

**Figure 1.**
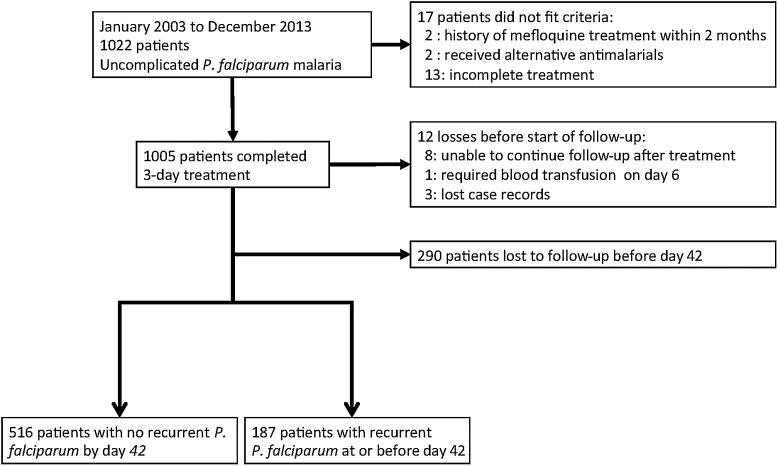
Patient flow diagram after 3-day treatment with mefloquine plus artesunate.

Between January 2003 and December 2013, 1022 patients were recruited, and 1005 remained in the study (Figure 1). Overall, 290 (28.8%) patients did not complete the full 42 days but were included in survival analyses. There were significant changes in gender ratio, age, duration of symptoms, and admission temperatures over the recruitment period, with an increasing proportion of older febrile male patients over time (Supplementary Table 1). The proportion of patients with fever at admission (tympanic temperature ≥37.5°C) rose significantly from 26.2% in 2003 to 65.4% in 2013 (*P* = .005). There was also an increase in the median duration of symptoms before presentation from 2 days in 2003 to 3 days in 2013 (*P* = .04).

### Clinical Responses

MAS3 was well tolerated and cleared the clinical symptoms rapidly. By 24 hours 84.3% (95% confidence interval [CI], 81.6, 86.7) and by 72 hours 98.0% (95% CI, 95.2, 99.1) of patients were afebrile. There was no change in fever clearance times over the study period. The mean fractional reduction in hematocrit from baseline to day 7 did not differ significantly by year (test for trend *P* = .08). Risk factors associated with anemia (hematocrit <30%) on admission were pretreatment gametocytemia (adjusted odds ratio [aOR], 9.43; 95% CI, 5.45, 16.34; *P* < .0001), age <13 years (aOR 3.77; 95% CI, 2.17, 6.56; *P* < .0001), and female gender (aOR 2.18; 95% CI, 1.28, 3.72; *P* = .004). No patient in the cohort developed severe malaria, and there were no deaths.

### *K13* Sequencing

**Figure 2.**
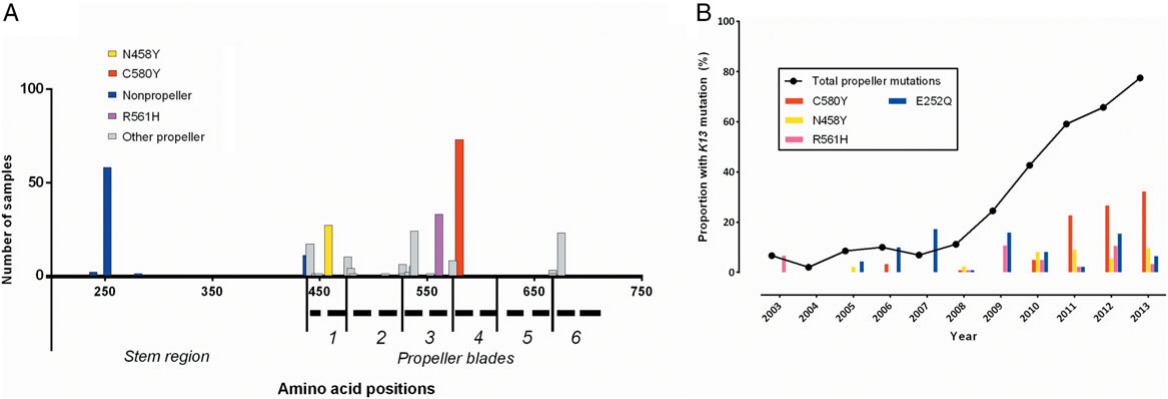
*A*, Primary amino acid positions and frequencies of *K13* mutations. (*B*) Annual proportions of *K13* propeller mutations (summed) and 4 individual genotypes (for which n > 25).

**Figure 3.**
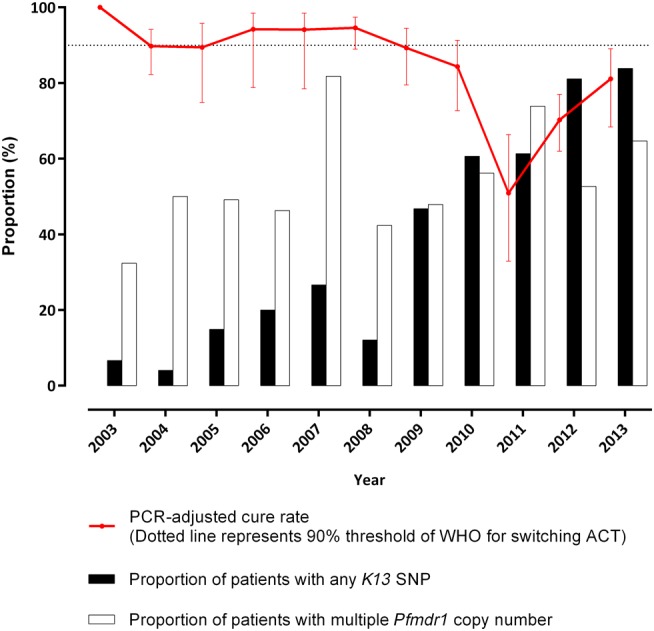
Annual proportions of day 42 polymerase chain reaction (PCR)–adjusted cure rates, summed *K13* mutations, and amplified *Pfmdr1*. Abbreviations: ACT, artemisinin-based combination treatment; SNP, single-nucleotide polymorphism; WHO, World Health Organization.

*K13* sequences from 699 (68.5%) admission parasite isolates and 112 (59.9%) recurrent isolates were analyzed (Supplementary Table 2). In the admission infections, 24 different nonsynonymous polymorphisms were detected, including 20 in the propeller region (Figure 2 and Supplementary Table 3). No sample had more than 1 mutation. The most frequent mutations were C580Y (10.4%) and E252Q (8.3%). The proportion of infections caused by isolates with any *K13* polymorphism increased from 6.7% (1/15; 95% CI, .2, 31.9) in 2003 to 83.9% (52/62; 95% CI, 72.3, 92.0) in 2013 (*P* < .001; Figure 3). During the period 2005–2009, the E252Q mutation was most common, but from 2010 the *K13* propeller mutations (notably C580Y) predominated (Figure 2*A* and 2*B*). More recurrent isolates had *K13* mutations (81/112 [72.3%]) compared with admission isolates (314/699 [44.9%]; *P* < .0001). In the recurrent isolates, more recrudescent isolates also had *K13* mutant alleles compared with reinfections (65/76 [85.5%] vs 12/24 [50%]; *P* < .001).

### *Pfmdr1* Copy Number

*Pfmdr1* copy numbers were measured in 726 (71.4%) admission isolates and 65 (34.8%) recurrent isolates (Supplementary Table 2). The proportion of infections caused by parasites with multiple (>1) *Pfmdr1* copies on admission doubled from 32.4% (95% CI, 17.4, 50.5) in 2003 to 64.7% (95% CI, 46.5, 80.3) in 2013 (*P* = .031; Figure 3). There was no significant difference in the distribution of *Pfmdr1* copy number with gender or age. Significantly more recurrent isolates had multiple copies of *Pfmdr1* 53/65 (81.5%) compared with admission isolates 377/726 (51.9%; *P* < .001). Among these recurrent isolates, more recrudescent isolates also had multiple copies of *Pfmdr1* compared with reinfections (38/42 [90.5%] vs 8/13 [61.5%]; *P* = .002).

Our study did not characterize the single nucleotide polymorphisms of the *Pfmdr1* gene because these relevant single-nucleotide polymorphisms (SNPs) are rare in Thailand [27] and mefloquine resistance is driven by copy number changes on a *Pfmdr1* wild-type background [4, 28–30]. Isolates carrying wild-type *K13* were associated with single-copy *Pfmdr1*, while *K13* mutations were associated with amplified *Pfmdr1* (Fisher exact test, *P* < .001) (Supplementary Figure 1).

The success rate for genotyping of *K13* and *Pfmdr1* was lower in recurrent infections because of significantly lower parasitemia (*P* = .0004) and thus much lower parasite DNA concentrations.

### Parasite Clearance

Clearance data were available for 957 patients (95.2%). There was a significant increase in the proportion of patients who were parasitemic at day 3 (*P* < .001, test for trend; Table 1). Multivariate analysis showed that mutation in the *K13* gene was the strongest risk factor for day 3 positivity, with later year of treatment, higher parasitemia, higher hematocrit, and fever on admission (but not *Pfmdr1* amplification) as independent risk factors (Table 2). *K13* propeller mutations were stronger predictors of day 3 positivity, and the 3 most common propeller mutations (C580Y, N458Y, and R561H) were each significantly associated with day 3 positivity (Table 2).

**Table 1.** Parasitological Responses to Mefloquine–Artesunate by Year

Years	2003	2004	2005	2006	2007	2008	2009	2010	2011	2012	2013
Completed treatment (n)	42	143	69	49	49	164	112	84	53	164	76
Proportion of day 3 blood slide positive (%)^a^	2/42 (4.8)	2/137 (1.5)	4/68 (5.9)	2/48 (4.2)	0/47 (0)	9/154 (5.8)	20/105 (19.0)	13/74 (17.6)	10/49 (20.4)	54/157 (34.4)	29/76 (38.2)
Proportion of day 4 blood slide positive (%)^a^	0/42 (0)	0/137 (0)	0/68 (0)	0/48 (0)	0/47 (0)	3/151 (2.0)	4/104 (3.8)	1/72 (1.4)	3/48 (6.3)	14/153 (9.2)	6/74 (8.1)
Unadjusted cure rate % (95% CI)	100	73.2 (62.4,81.3)	81.1 (65.4,90.2)	86.9 (67.6,95.1)	91.0 (74.6,97.0)	91.6 (84.6,95.5)	79.3 (67.0,87.5)	84.4 (72.7,91.3)	43.6 (23.9,61.9)	67.6 (59.3,74.6)	61.4 (47.0,73.0)
Polymerase chain reaction–adjusted cure rates (95% CI)	100	89.8 (82.3,94.2)	89.5 (74.9,95.8)	94.2 (78.8,98.5)	94.1 (78.5,98.5)	94.6 (89.0,97.4)	89.3 (79.5,94.5)	84.4 (72.7,91.3)	50.9 (32.9,66.4)	70.3 (62.0,77.0)	81.1 (68.4,89.1)
Days to recrudescence Median (range)	…	21 (15,24)	24 (14,49)	22 (22)	31 (28,34)	22 (19,26)	24 (14,37)	24.5 (17,38)	21 (14,41)	21 (7,36)	21 (21,35)
Days to reinfection Median (range)	…	37 (13,49)	40 (28,49)	42 (21,49)	41 (35,47)	37 (32,42)	36.5 (22,49)	…	45.5 (44,49)	30 (25,32)	21 (14,42)

Abbreviation: CI, confidence interval.

^a^ Excluding patients not attending who were positive at the previous visit.

**Table 2.** Predictors for Persistent Day 3 Asexual Parasitemia Following Mefloquine–Artesunate Treatment

Risk Factor	Adjusted Odds Ratio	95% Confidence Interval	*P* Value
Hematocrit on admission	1.04	1.00, 1.09	.04
Year of recruitment	1.14	1.02, 1.27	.02
Log parasitemia on admission	1.42	1.20, 1.68	<.001
Fever (threshold of 37.5°C on admission)	2.50	1.50, 4.18	<.001
Any *K13* mutant	6.59	3.53, 12.30	<.001
Subsets of *K13* mutants^a^			
Any *K13* propeller mutant	9.60	4.86, 18.95	<.001
Isolates with E252Q	2.25	.91, 5.58	.08
Isolates with C580Y	7.61	3.42, 16.95	<.001
Isolates with N458Y	8.75	3.10, 24.75	<.001
Isolates with R561H	14.98	5.35, 41.92	<.001

The analysis of *K13* mutant subsets produced similar (within 10%) adjusted odds ratios for fever, year of recruitment, parasitemia, and hematocrit compared with those obtained for any *K13* mutant.

^a^ Compared with wild type.

### Cure Rates

Of the 186 patients with recurrent *P. falciparum* infections, there were 117 (62.9%) recrudescences and 53 (28.5%) reinfections, with 2 indeterminate results, 1 amplification failure, and 13 missing samples. PCR-adjusted parasitological efficacy at day 42 remained above or close to 90% from 2003 to 2009 but declined sharply thereafter (test for trend, *P* < .001; Table 1, Figure 3). There was a similar trend for recurrence (PCR unadjusted) rate. The lowest cure rates were recorded in 2011 when PCR-adjusted and unadjusted cure rates fell to 43.6% and 50.9%, respectively.

### Predictors for Recrudescence

In a Cox regression model, increased *Pfmdr1* copy number and *K13* mutation were significant independent predictors of falciparum malaria recrudescence (with a multiplicative effect when in combination) along with year of recruitment and age (Table 3). The risk of recrudescence was even higher for *K13* propeller mutations as a group and for the 3 most common individual propeller mutations (C580Y, N458Y, and R561H; Table 3).

**Table 3.** Predictors for *Plasmodium falciparum* Recrudescence by Day 42 Following Mefloquine–Artesunate

Risk Factor	Adjusted Hazard Ratio	95% Confidence Interval	*P* Value
Age	0.97	.95, .99	.003
Year of recruitment	1.20	1.10, 1.44	.001
Multiple *Pfmdr1* copy number (>1)	2.68	1.52, 4.75	.001
Any *K13* mutant	3.84	1.77, 8.36	.001
Subsets of *K13* mutants^a^			
Any *K13* propeller mutant	4.76	2.11, 10.75	<.001
*K13* E252Q	2.83	1.03, 7.75	.04
*K13* C580Y	5.04	2.00, 12.75	.001
*K13* R561H	5.88	2.24, 15.40	<.001
*K13* N458Y	7.20	2.56, 20.24	<.001
Compound genotypes^b^			
Multiple *Pfmdr1* + wild type *K13*	3.27	.84, 12.65	.09
Single *Pfmdr1* + *K13* propeller mutant	5.73	1.54, 21.26	.009
Multiple *Pfmdr1* + *K13* propeller mutant	14.05	3.99, 49.48	<.001

The analysis of *K13* mutant subsets and compound *K13/Pfmdr1* genotypes produced similar (within 10%) adjusted hazard ratios for age and year of recruitment compared with the main model.

^a^ Compared with wild type.

^b^ Compared with single *Pfmdr1* copy number and wild-type *K13*.

**Figure 4.**
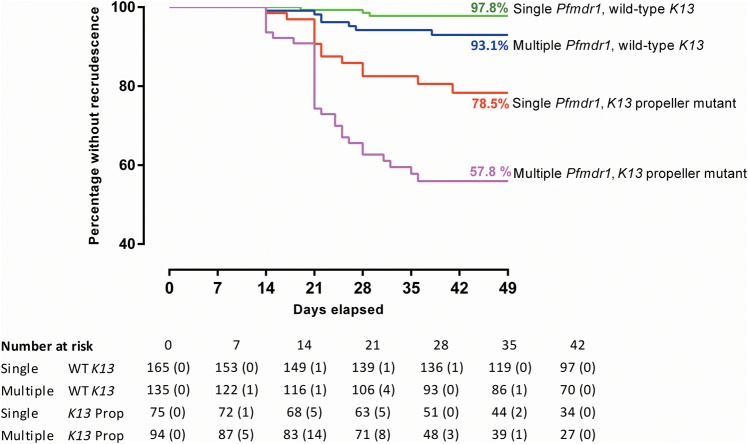
Kaplan–Meier curves showing cumulative proportions of patients free from recrudescence stratified by presence of *Pfmdr1* copy number and *K13* genotype. The numbers in parentheses are the recrudescences during the indicated time. *K13* Prop refers to samples with mutation in the *K13* propeller.

Cure rates were highest for infections with isolates with single-copy *Pfmdr1* gene and wild-type *K13* (97.8%) and lowest in patients with multiple copies of *Pfmdr1* and any *K13* propeller SNP (57.8%; Table 4, Figures 4 and Supplementary Figure 2). Cure rates declined markedly in recent years as the prevalence of *K13* mutations rose (Supplementary Figure 2).

**Table 4.** Day 42 Polymerase Chain Reaction–Adjusted Adequate Clinical and Parasitological Response Rates Determined by Survival Analysis for 4 Compound Genotypes

Genotype	Cure Rate	95% Confidence Interval
Single *Pfmdr1* + wild-type *K13*	97.8	(93.3, 99.3)
Multiple *Pfmdr1* + wild-type *K13*	93.1	(86.0, 96.7)
Single *Pfmdr1* + *K13* propeller mutant	78.5	(65.6, 87.0)
Multiple *Pfmdr1* + *K13* propeller mutant	57.8	(45.4, 68.3)

The PAFs (equivalent to the percentage reduction in recrudescent infections that would occur if these mutations were not present) for *K13* and *Pfmdr1* amplification were 69.0 and 41.9%, respectively. The PAF for the 2 factors in combination was 82%. Hence these 2 factors alone explain almost entirely the increased ACT treatment failure rate observed in this population.

### Pre- and Post-treatment Gametocytemia

The proportions of patients presenting with pre-treatment gametocytes or with post-treatment gametocytemia and the associated risk factors are shown in the Supplementary Materials (Supplementary Tables 1, 1.1 and 1.2). There was no association between the presence of *K13* propeller mutations (aOR 1.02; 95% CI, .47, 2.19) or multiple copies of *Pfmdr1* (aOR 1.28; 95% CI, .75, 2.17) and gametocytemia, either at admission or during follow-up.

## DISCUSSION

MAS3 for the treatment of uncomplicated *P. falciparum* malaria has had a remarkable therapeutic longevity and a dramatic impact on morbidity and mortality on the Thailand–Myanmar border. When artesunate was introduced in 1991, mefloquine was a failing drug, the incidence of falciparum malaria was rising, and most parasite isolates had multiple copies of *Pfmdr1* associated with mefloquine resistance [4]. After deployment of this ACT in 1994, mefloquine recovered its efficacy, and less fit isolates with multiple *Pfmdr1* copies were replaced by single copy–containing isolates [31]. High cure rates were then sustained for more than a decade [22, 32]. In 2006, 53% of patients presenting with uncomplicated falciparum malaria had infections with multiple copies of *Pfmdr1* [22]. The rise in prevalence of *P. falciparum* parasites with *K13* propeller mutations was the definitive event that led to the demise of MAS3. The temporal sequence of *K13* selection is informative. The E252Q mutation (which is located on the stem region of the *K13* gene; Figure 2*A*), associated with moderate slowing of parasite clearance [15], predominated before 2010. Then parasites with the E252Q mutation were progressively overtaken by parasites with *K13* propeller mutations, conferring greater reductions in parasite clearance rates. The main propeller mutation was C580Y, a polymorphism common in Cambodia [14, 15]. The E252Q mutation was associated with a moderate increase in risk of treatment failure, suggesting that the selective advantage of the *K13* mutation is proportional to the degree of parasite clearance prolongation. It is possible that genetic changes outside the *K13* locus have contributed to the emergence of artemisinin resistance and the selection of *K13* mutations [20]. It remains to be seen whether parasite genotypes with even slower clearance will become established while ACTs remain the mainstay of antimalarial treatment.

The large body of data presented here strongly support the hypothesis that there is a substantial impact of *K13* mutations on treatment failure. *K13* mutations in admission samples are associated with a failure rate of 21.5%. In combination with multiple copies of *Pfmdr1*, this rises to 42.2%. The sharp decline in MAS3 cure rates temporally corresponds with the emergence of *K13* propeller mutant isolates on a long-standing genetic background of *Pfmdr1* amplification. The proportion of treatment failures that can be attributed to *K13* mutations and multiple copies of *Pfmdr1* (ie, PAF) is 82%. Hence, these 2 factors alone explain the majority of increase in treatment failure observed. Other parasite genetic factors that our study was not designed to detect or changes in the demographics of the patient population may have contributed to the 18% of unexplained variation in treatment failure.

We highlight 3 additional features of particular interest. First, *K13* mutations and *Pfmdr1* amplification have a multiplicative (rather than an additive) effect on risk of treatment failure. Synergy between these 2 resistance determinants may help to explain why failure rate declined precipitously in 2009—parasites carrying both markers only became common as *K13* mutations rose in frequency. Second, in contrast to the 2 Cambodian studies, we observed multiple *K13* mutations in this study. These data demonstrate significant heterogeneity in the impact of different *K13* mutations on treatment failure. A mutation outside the *K13* propeller (E252Q) increased treatment failure relative to wild-type parasites, but the 3 common propeller mutations had a much greater effect (Table 3). These data suggest that surveillance for emergence of *K13* mutations should be expanded to include *K13* regions outside the propeller domain. Third, 2 studies [15, 19] provide evidence that gametocyte carriage is elevated in parasites bearing *K13* mutations, leading to the suggestion that such parasites may have a transmission advantage. In contrast, in this large study from a single location, we observed no association between gametocyte carriage and *K13* mutations.

What are the implications of these data for the therapeutic life span of ACTs in Southeast Asia and beyond? The most widely deployed ACT is the coformulation of artemether and lumefantrine (AL), which has a resistance mechanism that is similar to that of mefloquine, involving *Pfmdr1* amplification [29, 33, 34]. In most areas where AL is used, there is no evidence for resistance to either component. However, in Myanmar where AL has recently been introduced, *K13* propeller mutants and *Pfmdr1* amplification are widespread [28, 35–38]. The therapeutic lifetime of AL in Myanmar and other areas with a high prevalence of *K13* mutations may be relatively short.

ACTs containing an alternative partner drug should provide relief in the short term. In 2012 the first-line treatment of uncomplicated *P. falciparum* malaria in our treatment centers on the Thailand–Myanmar border was changed from MAS3 to DHA–piperaquine, which is currently highly efficacious in this area but relies increasingly on the piperaquine component. The recent emergence of piperaquine resistance in Cambodia [39] and associated rising failure rates with DHA–piperaquine [19, 21, 40] also cast doubt over the long-term future of this combination. Alternatives are needed desperately. With new antimalarials still years from deployment, there is an urgent need to eliminate *P. falciparum* from the area before the recent and substantial gains in malaria control are reversed.

## Supplementary Data

Supplementary materials are available at http://cid.oxfordjournals.org. Consisting of data provided by the author to benefit the reader, the posted materials are not copyedited and are the sole responsibility of the author, so questions or comments should be addressed to the author.

Supplementary Data

## References

[1] Nosten F, Imvithaya S, Vincenti M et al Malaria on the Thai-Burmese border: treatment of 5192 patients with mefloquine-sulfadoxine-pyrimethamine. Bull World Health Organ 1987; 65:891–6. 3325187 PMC2491097

[2] Nosten F, Luxemburger C, ter Kuile FO et al Treatment of multidrug-resistant *Plasmodium falciparum* malaria with 3-day artesunate-mefloquine combination. J Infect Dis 1994; 170:971–7. 7930743 10.1093/infdis/170.4.971

[3] Nosten F, ter Kuile F, Chongsuphajaisiddhi T et al Mefloquine-resistant falciparum malaria on the Thai-Burmese border. Lancet 1991; 337:1140–3. 1674024 10.1016/0140-6736(91)92798-7

[4] Price RN, Uhlemann AC, Brockman A et al Mefloquine resistance in *Plasmodium falciparum* and increased pfmdr1 gene copy number. Lancet 2004; 364:438–47. 15288742 10.1016/S0140-6736(04)16767-6 PMC4337987

[5] ter Kuile FO, Nosten F, Thieren M et al High-dose mefloquine in the treatment of multidrug-resistant falciparum malaria. J Infect Dis 1992; 166:1393–400. 1431257 10.1093/infdis/166.6.1393

[6] Price RN, Nosten F, Luxemburger C et al Effects of artemisinin derivatives on malaria transmissibility. Lancet 1996; 347:1654–8. 8642959 10.1016/s0140-6736(96)91488-9

[7] Nosten F, van Vugt M, Price R et al Effects of artesunate-mefloquine combination on incidence of *Plasmodium falciparum* malaria and mefloquine resistance in western Thailand: a prospective study. Lancet 2000; 356:297–302. 11071185 10.1016/s0140-6736(00)02505-8

[8] Carrara VI, Lwin KM, Phyo AP et al Malaria burden and artemisinin resistance in the mobile and migrant population on the Thai-Myanmar border, 1999–2011: an observational study. PLoS Med 2013; 10:e1001398. 23472056 10.1371/journal.pmed.1001398 PMC3589269

[9] Nkhoma SC, Nair S, Al-Saai S et al Population genetic correlates of declining transmission in a human pathogen. Mol Ecol 2013; 22:273–85. 23121253 10.1111/mec.12099 PMC3537863

[10] Noedl H, Se Y, Sriwichai S et al Artemisinin resistance in Cambodia: a clinical trial designed to address an emerging problem in Southeast Asia. Clin Infect Dis 2010; 51:e82–9. 21028985 10.1086/657120

[11] Dondorp AM, Nosten F, Yi P et al Artemisinin resistance in *Plasmodium falciparum* malaria. N Engl J Med 2009; 361:455–67. 19641202 10.1056/NEJMoa0808859 PMC3495232

[12] Phyo AP, Nkhoma S, Stepniewska K et al Emergence of artemisinin-resistant malaria on the western border of Thailand: a longitudinal study. Lancet 2012; 379:1960–6. 22484134 10.1016/S0140-6736(12)60484-X PMC3525980

[13] Cheeseman IH, Miller BA, Nair S et al A major genome region underlying artemisinin resistance in malaria. Science 2012; 336:79–82. 22491853 10.1126/science.1215966 PMC3355473

[14] Ariey F, Witkowski B, Amaratunga C et al A molecular marker of artemisinin-resistant *Plasmodium falciparum* malaria. Nature 2014; 505:50–5. 24352242 10.1038/nature12876 PMC5007947

[15] Ashley EA, Dhorda M, Fairhurst RM et al Spread of artemisinin resistance in *Plasmodium falciparum* malaria. N Engl J Med 2014; 371:411–23. 25075834 10.1056/NEJMoa1314981 PMC4143591

[16] Ferreira PE, Culleton R, Gil JP, Meshnick SR Artemisinin resistance in *Plasmodium falciparum*: what is it really? Trends Parasitol 2013; 29:318–20. 23768531 10.1016/j.pt.2013.05.002

[17] Hastings IM, Kay K, Hodel EM How robust are malaria parasite clearance rates as indicators of drug effectiveness and resistance? Antimicrob Agents Chemother 2015; 59:6428–36. 26239987 10.1128/AAC.00481-15 PMC4576129

[18] Krishna S, Kremsner PG Antidogmatic approaches to artemisinin resistance: reappraisal as treatment failure with artemisinin combination therapy. Trends Parasitol 2013; 29:313–7. 23623760 10.1016/j.pt.2013.04.001

[19] Spring MD, Lin JT, Manning JE et al Dihydroartemisinin-piperaquine failure associated with a triple mutant including kelch13 C580Y in Cambodia: an observational cohort study. Lancet Infect Dis 2015; 15:683–91. 25877962 10.1016/S1473-3099(15)70049-6

[20] Miotto O, Amato R, Ashley EA et al Genetic architecture of artemisinin-resistant *Plasmodium falciparum*. Nat Genet 2015; 47:226–34. 25599401 10.1038/ng.3189 PMC4545236

[21] Amaratunga C, Lim P, Suon S et al Dihydroartemisinin–piperaquine resistance in *Plasmodium falciparum* malaria in Cambodia: a multisite prospective cohort study. Lancet Infect Dis 2016; 16:357–65. 26774243 10.1016/S1473-3099(15)00487-9 PMC4792715

[22] Carrara VI, Zwang J, Ashley EA et al Changes in the treatment responses to artesunate-mefloquine on the northwestern border of Thailand during 13 years of continuous deployment. PLoS One 2009; 4:e4551. 19234601 10.1371/journal.pone.0004551 PMC2641001

[23] Luxemburger C, Thwai KL, White NJ et al The epidemiology of malaria in a Karen population on the western border of Thailand. Trans R Soc Trop Med Hyg 1996; 90:105–11. 8761562 10.1016/s0035-9203(96)90102-9

[24] World Health Organization. Guidelines for the treatment of malaria. 2nd ed Geneva: World Health Organization, 2010.

[25] Ashley EA, Lwin KM, McGready R et al An open label randomized comparison of mefloquine-artesunate as separate tablets vs. a new co-formulated combination for the treatment of uncomplicated multidrug-resistant falciparum malaria in Thailand. Trop Med Int Health 2006; 11:1653–60. 17054744 10.1111/j.1365-3156.2006.01724.x

[26] Brockman A, Paul RE, Anderson TJ et al Application of genetic markers to the identification of recrudescent *Plasmodium falciparum* infections on the northwestern border of Thailand. Am J Trop Med Hyg 1999; 60:14–21. 9988316 10.4269/ajtmh.1999.60.14

[27] Anderson TJ, Nair S, Qin H et al Are transporter genes other than the chloroquine resistance locus (pfcrt) and multidrug resistance gene (pfmdr) associated with antimalarial drug resistance? Antimicrob Agents Chemother 2005; 49:2180–8. 15917511 10.1128/AAC.49.6.2180-2188.2005 PMC1140548

[28] Nair S, Nash D, Sudimack D et al Recurrent gene amplification and soft selective sweeps during evolution of multidrug resistance in malaria parasites. Mol Biol Evol 2007; 24:562–73. 17124182 10.1093/molbev/msl185

[29] Lim P, Alker AP, Khim N et al Pfmdr1 copy number and arteminisin derivatives combination therapy failure in falciparum malaria in Cambodia. Malar J 2009; 8:11. 19138391 10.1186/1475-2875-8-11 PMC2627910

[30] Muhamad P, Chaijaroenkul W, Phompradit P, Rueangweerayut R, Tippawangkosol P, Na-Bangchang K Polymorphic patterns of pfcrt and pfmdr1 in *Plasmodium falciparum* isolates along the Thai-Myanmar border. Asian Pac J Trop Biomed 2013; 3:931–5. 24093782 10.1016/S2221-1691(13)60181-1 PMC3805099

[31] Preechapornkul P, Imwong M, Chotivanich K et al *Plasmodium falciparum* pfmdr1 amplification, mefloquine resistance, and parasite fitness. Antimicrob Agents Chemother 2009; 53:1509–15. 19164150 10.1128/AAC.00241-08 PMC2663078

[32] Rueangweerayut R, Phyo AP, Uthaisin C et al Pyronaridine-artesunate versus mefloquine plus artesunate for malaria. N Engl J Med 2012; 366:1298–309. 22475593 10.1056/NEJMoa1007125

[33] Price RN, Uhlemann AC, van Vugt M et al Molecular and pharmacological determinants of the therapeutic response to artemether-lumefantrine in multidrug-resistant *Plasmodium falciparum* malaria. Clin Infect Dis 2006; 42:1570–7. 16652314 10.1086/503423 PMC4337983

[34] Venkatesan M, Gadalla NB, Stepniewska K et al Polymorphisms in *Plasmodium falciparum* chloroquine resistance transporter and multidrug resistance 1 genes: parasite risk factors that affect treatment outcomes for *P. falciparum* malaria after artemether-lumefantrine and artesunate-amodiaquine. Am J Trop Med Hyg 2014; 91:833–43. 25048375 10.4269/ajtmh.14-0031 PMC4183414

[35] Triglia T, Foote SJ, Kemp DJ, Cowman AF Amplification of the multidrug resistance gene pfmdr1 in *Plasmodium falciparum* has arisen as multiple independent events. Mol Cell Biol 1991; 11:5244–50. 1922044 10.1128/mcb.11.10.5244-5250.1991 PMC361573

[36] Tun KM, Imwong M, Lwin KM et al Spread of artemisinin-resistant *Plasmodium falciparum* in Myanmar: a cross-sectional survey of the K13 molecular marker. Lancet Infect Dis 2015; 15:415–21. 25704894 10.1016/S1473-3099(15)70032-0 PMC4374103

[37] Huang F, Takala-Harrison S, Jacob CG et al A Single mutation in K13 predominates in southern China and is associated with delayed clearance of *Plasmodium falciparum* following artemisinin treatment. J Infect Dis 2015; 212:1629–35. 25910630 10.1093/infdis/jiv249 PMC4621243

[38] Putaporntip C, Kuamsab N, Kosuwin R et al Natural selection of K13 mutants of *Plasmodium falciparum* in response to artemisinin combination therapies in Thailand. Clin Microbiol Infect 2016; 22:285e1–8. 26548510 10.1016/j.cmi.2015.10.027

[39] Chaorattanakawee S, Saunders DL, Sea D et al Ex vivo drug susceptibility testing and molecular profiling of clinical *Plasmodium falciparum* isolates from Cambodia from 2008 to 2013 suggest emerging piperaquine resistance. Antimicrob Agents Chemother 2015; 59:4631–43. 26014942 10.1128/AAC.00366-15 PMC4505231

[40] Leang R, Taylor WR, Bouth DM et al Evidence of *Plasmodium falciparum* malaria multidrug resistance to artemisinin and piperaquine in Western Cambodia: dihydroartemisinin-piperaquine open-label multicenter clinical assessment. Antimicrob Agents Chemother 2015; 59:4719–26. 26014949 10.1128/AAC.00835-15 PMC4505193

