# Supplementary material for: Declining Efficacy of Artemisinin Combination Therapy Against P. Falciparum Malaria on the Thai–Myanmar Border (2003–2013): The Role of Parasite Genetic Factors
Source: Clin Infect Dis. 2016 Jun 16;63(6):784–91. doi: 10.1093/cid/ciw388 (PMC4996140; doi:10.1093/cid/ciw388)
Supplement: Supplementary Data [file supp_63_6_784__index.html]

Declining efficacy of artemisinin combination therapy against P. falciparum malaria on the Thai-Myanmar border (2003-2013): the role of parasite genetic factors — Declining Efficacy of Artemisinin Combination Therapy Against P. Falciparum Malaria on the Thai–Myanmar Border (2003–2013): The Role of Parasite Genetic Factors — Declining Efficacy of Artemisinin Combination Therapy Against P. Falciparum Malaria on the Thai–Myanmar Border (2003–2013): The Role of Parasite Genetic Factors — Supplementary Data 

# Declining Efficacy of Artemisinin Combination Therapy Against *P. Falciparum* Malaria on the Thai–Myanmar Border (2003–2013): The Role of Parasite Genetic Factors

## Supplementary Data

Supplementary Data

- Supplementary Data - Docx file
- Supplementary Figure 1 - jpg file
- Supplementary Figure 2 - jpg file
- Supplementary Tables - pdf file
