# Supplementary material for: Declining Efficacy of Artemisinin Combination Therapy Against P. Falciparum Malaria on the Thai–Myanmar Border (2003–2013): The Role of Parasite Genetic Factors
Source: Clin Infect Dis. 2016 Jun 16;63(6):784–91. doi: 10.1093/cid/ciw388 (PMC4996140; doi:10.1093/cid/ciw388)
Supplement: Supplementary Data [file supp_ciw388_ciw388supp.docx]

# Supplementary Methods

## Genotyping procedures to identify parasite populations using MSP-1, MSP-2, GLURP

We adapted Recommended Genotyping Procedures (RGPs) to identify parasite populations by the World Health Organization, 2007.

N.B. Recurrent infection=Reinfection + Recrudescence

## Copy number variation in Pfmdr1

## DNA from blood spots was extracted using the QIAamp DNA Blood Mini Kit (Qiagen) and eluted into a final volume of 100µl. 1 µl of the DNA eluent was used for each quantitative PCR reaction for gene copy number measurements. These were carried out in a final volume of 20ul containing 1 x concentration of the Power SYBR Green Master Mix (Applied Biosystems^TM^) and 125nM of oligonucleotide primers using the 7500 FAST REAL-TIME PCR system thermocycler (ABI, USA) with the following programme: initial activation 56^°^C for 2 minutes and 90^°^C for 10 min followed by amplification via 40 cycles of 95^°^C for 30 seconds, 55^°^C for 1 min and 68^°^C for 1 min. The pfmdr1 copy number was measured with respect to actin, a single copy gene in the P. falciparum genome. For each sample two identical qPCR reactions were carried out in parallel using two sets of oligonucleotide primers: Pfmdr1-F: CAAGTGAGTTCAGGAATTGGT and Pfmdr1-R: GCCTCTTCTATAATGGACATGG for pfmdr1 (gene ID# PF3D7_0523000) and Pf-B-actin-F: GTTACGAATTGGGATGATATGG and Pf-B-actin-R: CTCCACTATCTAACACAATACC for actin (gene ID# PF3D7_1246200). At the end of each reaction, Cycle threshold (Ct) was adjusted manually to best capture the kinetic PCR profile. The final pfmdr1 copy number was calculated as 2^ΔCT^ where ∆Ct = Ct(pfmdr1) - Ct(actin), rounded to the nearest integer.

As a quality control measurement, repeated assay of the 3d7 reference laboratory isolate (known to have one copy of Pfmdr1) showed a median Pfmdr1 copy number of 1.039 (SD = 0.537; n=32).

## Genetic markers of artemisinin resistance

The primary hypothesis was to examine the association between mutations in the *K13* propeller region, (amino acids 441 onwards) although the less well studied ‘stem’ region of the protein (amino acids 211 – 302) was also investigated.

The *K13* propeller region (1725980-1726940bp, positions 419-707) was amplified by using two primer sets: fragment 1 (1725980-1726520bp, pos 419-570): F- ATCTAGGGGTATTCAAAGG, R- CCAAAAGATTTAAGTGAAAG; fragment 2 (1726400-1726940bp, pos 545-707): F-CTGCCATTCATTTGTATCT, R- GGATATGATGGCTCTTCTA. We also amplified the 5' region: fragment 3 (1725380-1725680bp, pos 211-302) using primers F- TGAAAATATGGTAGGTGATT and R- ATCGTTTCCTATGTTCTTCT. PCR products were treated with Exo-SAP-IT (GE Healthcare), and sequenced directly in both directions using the BigDye Terminator v3.1 cycle sequencing kit (Applied Biosystems, Inc., Foster City, CA). BigDye products were cleaned using the BigDye XTerminator Purification kit (ABI, USA) and then run on a ABI 3730 capillary sequencer. The data were aligned and analysed using SeqScape version 2.7.
