# Supplementary figures and images for: Declining Efficacy of Artemisinin Combination Therapy Against P. Falciparum Malaria on the Thai–Myanmar Border (2003–2013): The Role of Parasite Genetic Factors
Source: Clin Infect Dis. 2016 Jun 16;63(6):784–91. doi: 10.1093/cid/ciw388 (PMC4996140; doi:10.1093/cid/ciw388)

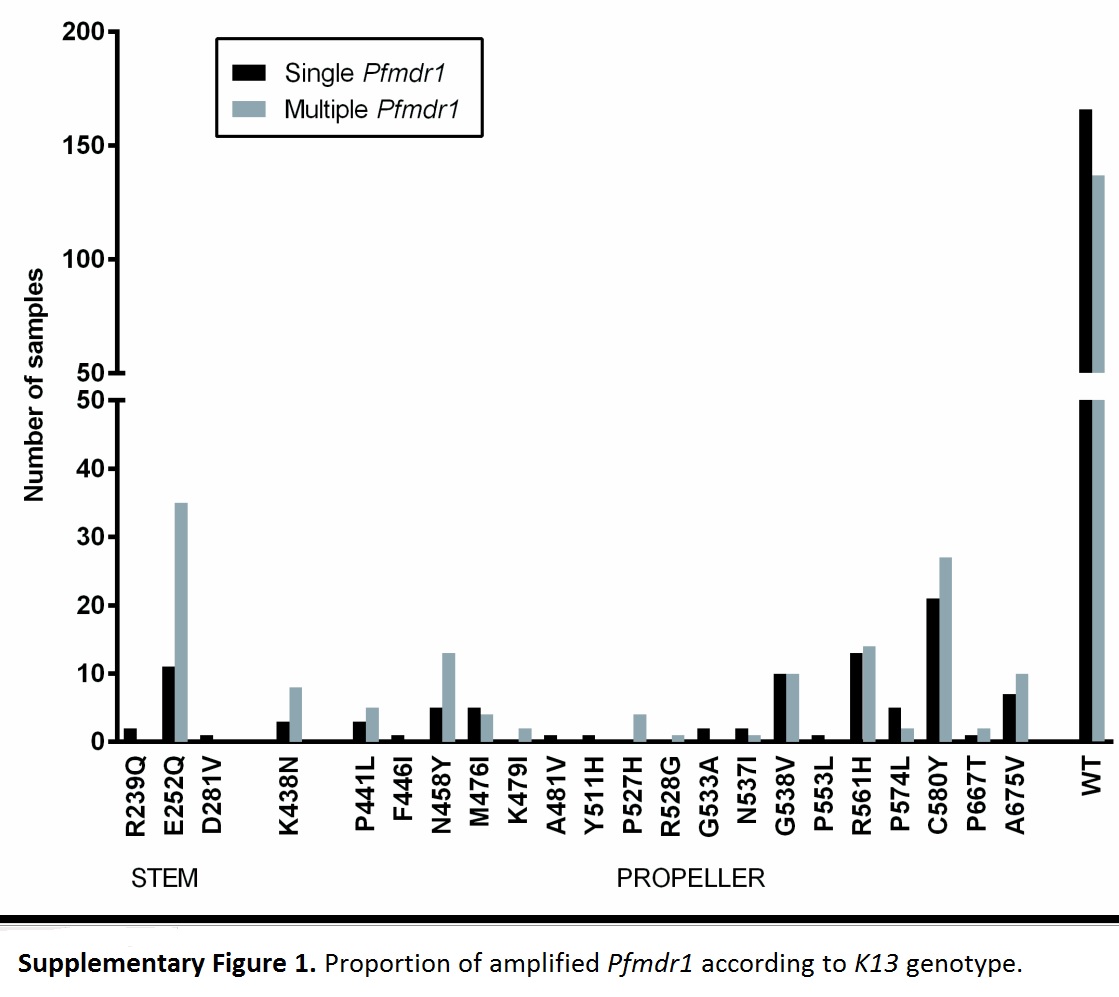

Supplement: Supplementary Data [file supp_ciw388_ciw388supp_fig1.jpg]

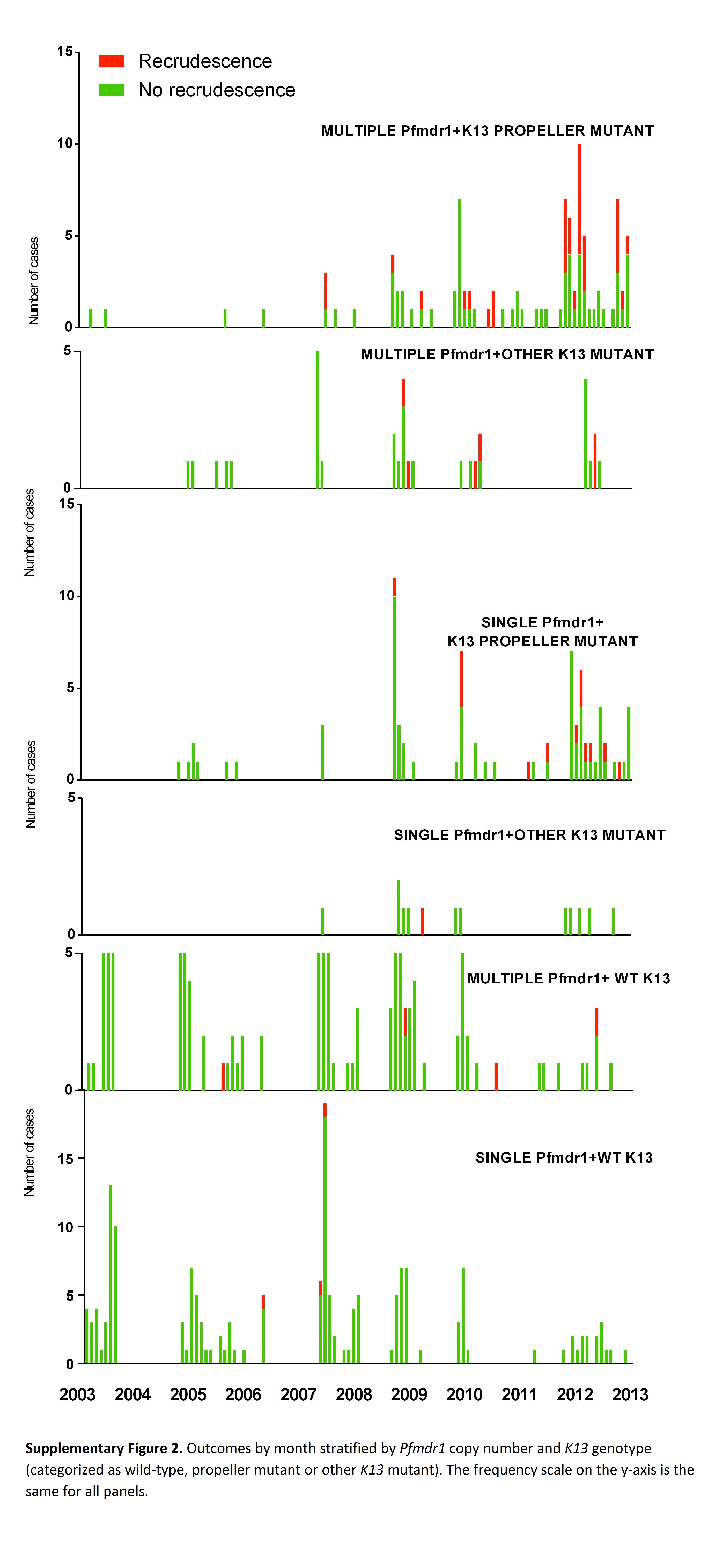

Supplement: Supplementary Data [file supp_ciw388_ciw388supp_fig2.jpg]
