# Supplementary material for: Declining Efficacy of Artemisinin Combination Therapy Against P. Falciparum Malaria on the Thai–Myanmar Border (2003–2013): The Role of Parasite Genetic Factors
Source: Clin Infect Dis. 2016 Jun 16;63(6):784–91. doi: 10.1093/cid/ciw388 (PMC4996140; doi:10.1093/cid/ciw388)
Supplement: Supplementary Data [file supp_ciw388_ciw388supp_tables.pdf]

Supplementary Table 1: Baseline demographic and clinical data

Years	2003	2004	2005	2006	2007	2008	2009	2010	2011	2012	2013	Total
Recruited n (%)	42 (4.1)	145 (14.2)	70 (6.9)	49 (4.8)	50 (4.9)	165 (16.1)	115 (11.3)	86 (8.4)	54 (5.3)	168 (16.4)	78 (7.6)	1022 (100)
Male n (%)	23 (54.8)	83 (57.2)	47 (67.1)	34 (69.4)	42 (84.0)	130 (78.8)	97 (84.4)	64 (74.4)	43 (79.6)	139 (82.7)	55 (70.5)	757 (73)
Age, years												
Mean (sd)	22.0 (14.4)	23.3 (15.2)	24.5 (12.2)	29.6 (12.4)	32.3 (14.7)	26.9 (12.7)	29.3 (12.5)	28.7 (12.5)	27.2 (13.2)	26.5 (13.3)	26.7 (15.4)	26.8 (13.7)
<5, n (%)	3 (7.1)	8 (5.5)	0 (0)	0 (0)	0 (0)	4 (2.4)	1 (0.9)	1 (1.2)	0 (0)	1 (0.6)	0 (0)	18 (1.8)
5-15, n (%)	14 (33.3)	53 (36.6)	16 (22.9)	9 (18.4)	6 (12.0)	22 (13.3)	14 (12.2)	11 (18.8)	13 (24.1)	43 (25.6)	24 (30.8)	225 (22.0)
>15, n (%)	25 (59.6)	84 (57.9)	54 (77.1)	40 (81.6)	44 (88.0)	139 (84.2)	100 (87.0)	74 (86.0)	41 (75.9)	124 (73.8)	54 (69.2)	779 (76.2)
Weight, kg												
Mean (sd)	38.9 (14.4)	40.0 (14.8)	45.9 (13.2)	48.0 (12.5)	50.6 (11.7)	48.1 (12.2)	48.9 (10.5)	50.1 (11.8)	48.2 (12.0)	46.6 (11.7)	43.6 (13.5)	46.2 (13.0)
**Clinical Profiles**												
Fever (Temp≥37.5) n (%)	11 (26.2)	59 (44.0)	43 (61.4)	24 (49.0)	15 (33.3)	48 (39.7)	41 (54.7)	44 (54.3)	36 (66.7)	107 (63.7)	51 (65.4)	479 (52.2)
Mean temperature (sd)	36.9 (1.1)	37.5 (1.2)	37.8 (1.0)	37.3 (0.9)	37.2 (1.0)	37.4 (1.1)	37.7 (0.9)	37.7 (0.9)	37.8 (0.8)	37.8 (1.0)	37.8 (0.9)	37.6 (1.0)
Duration of fever (days)	2	2	2	2	2	2	2	2	2	3	2	2
Median (range)	(1, 4)	(0, 10)	(1, 7)	(1, 7)	(1, 9)	(1, 10)	(0, 10)	(0, 7)	(0, 7)	(1, 11)	(1, 7)	(1, 7)
Duration of symptoms (days)												
Median (range)	2 (1, 4)	3 (0, 10)	2 (1, 7)	2 (1, 7)	3 (1, 9)	3 (0, 11)	2 (1, 10)	2 (1, 7)	3 (1, 7)	3 (1, 11)	3 (1, 10)	3 (0, 11)
Mean Haematocrit (sd)	37.3 (5.0)	34.8 (6.1)	38.0 (5.1)	38.1 (5.8)	38.6 (4.6)	39.7 (6.2)	39.7 (6.5)	39.4 (0.7)	39.0 (0.9)	40.6 (0.4)	38.2 (0.7)	38.6 (0.2)
Parasite density												
Geometric mean (per µL)	10646	7903	10625	8903	5101	9174	9367	11437	11291	12556	11672	9849
Min	80	48	128	160	64	96	208	448	128	64	112	48
Max	114296	190912	142430	206612	171444	160266	170816	179357	174082	214776	178478	214776
Pre-treatment Gametocytemia	7.1	10.3	8.6	12.2	10.0	6.8	14.9	10.6	7.4	13.1	9.0	10.3
%	1.5, 19.5	5.9, 16.5	3.2, 17.7	4.6, 24.8	3.3, 21.8	3.4, 11.8	8.9, 22.8	5.0, 19.2	2.1, 17.9	8.4, 19.2	3.7, 17.6	6.1, 18.5
Post-treatment Gametocytemia	9.5	12.6	13.0	14.3	10.2	18.6	17.9	17.9	17.3	17.1	13.3	15.5
%	2.7, 22.6	7.6, 19.2	6.1, 23.3	5.9, 27.2	3.4, 22.2	12.9, 25.5	11.2, 26.2	10.4, 27.7	8.2, 30.3	11.7, 23.7	6.6, 23.2	13.3, 17.9

Supplementary Table 1.1: Predictors for Pre-treatment gametocytemia

Risk factor	aOR	95% CI	*P*
Hematocrit on admission	0.83	0.80, 0.87	<0.001
Year of recruitment	1.09	1.02, 1.18	0.01
Duration of symptoms (days)	1.13	1.04, 1.23	0.004

Supplementary Table 1.2: Predictors for Post-treatment gametocytemia

Risk factor	aOR	95% CI	*P*
Pre-treatment gametocytemia	23.5	13.6, 40.5	<0.001
Hematocrit on admission	0.95	0.92, 0.99	0.007

Supplementary Table 2: Trends in molecular markers over the duration of the study*

Years	2003	2004	2005	2006	2007	2008	2009	2010	2011	2012	2013	Total
**Pfmdr1 copy number**												
(n) admission isolates	34	108	59	41	33	102	109	73	23	110	34	726
Pfmdr1 CN = 1 n (%)	23 (67.6)	54 (50.0)	30 (50.8)	22 (53.7)	6 (18.2)	59 (57.8)	53 (48.6)	32 (43.8)	6 (26.1)	52 (47.3)	12 (35.3)	349 (48.1)
Pfmdr1 CN = 2 n (%)	7 (20.6)	18 (16.7)	19 (32.2)	7 (17.1)	11 (33.3)	16 (15.7)	26 (23.9)	23 (31.5)	8 (34.8)	29 (26.4)	6 (17.6)	170 (23.4)
Pfmdr1 CN = 3 n (%)	1 (2.9)	13 (12.0)	7 (11.9)	3 (7.3)	3 (9.1)	7 (6.9)	21 (19.3)	12 (16.4)	7 (30.4)	18 (16.4)	7 (20.6)	99 (13.6)
Pfmdr1 CN >3 n (%)	3 (8.8)	23 (21.3)	3 (5.1)	9 (22.0)	13 (39.4)	20 (19.6)	9 (8.3)	6 (8.2)	2 (8.7)	11 (10.0)	9 (26.5)	108 (14.9)
(n) cases at recurrence	0	0	0	5	2	8	10	7	2	15	16	65
Pfmdr1 CN = 1 n (%)	-	-	-	1 (20.0)	2 (100)	3 (37.5)	0 (0)	0 (0)	0 (0)	2 (13.3)	4 (25.0)	12 (18.5)
Pfmdr1 CN = 2 n (%)	-	-	-	2 (40.0)	0 (50.0)	3 (37.5)	2 (20.0)	2 (28.6)	0 (0)	6 (40.0)	4 (25.0)	19 (29.2)
Pfmdr1 CN = 3 n (%)	-	-	-	0 (0)	0 (0)	1 (12.5)	3 (30.0)	4 (57.1)	2 (100)	5 (33.3)	7 (43.8)	22 (33.9)
Pfmdr1 CN >3 n (%)	-	-	-	2 (40.0)	0 (0)	1 (12.5)	5 (50.0)	1 (14.3)	0 (0)	2 (13.3)	1 (6.3)	12 (18.5)
**K13 sequence**												
(n) admission isolates	15	49	47	30	30	124	94	61	44	143	62	699
Wild type isolates n (%)	14 (93.3)	48 (98.0)	40 (85.1)	24 (80.0)	22 (73.3)	109 (87.9)	50 (53.2)	24 (39.3)	17 (38.6)	27 (18.9)	10 (16.1)	384 (54.9)
Isolates with any SNP n (%)	1 (6.7)	1 (2.0)	7 (14.9)	6 (20.0)	8 (26.7)	15 (12.1)	44 (46.8)	37 (60.7)	27 (61.4)	116 (81.1)	52 (83.9)	315 (45.1)
Isolates with C580Y n (%)	0 (0)	0 (0)	0 (0)	1 (3.3)	0 (0)	1 (0.8)	0 (0)	3 (4.9)	10 (22.7)	38 (26.6)	20 (32.3)	73 (10.4)
Isolates with E252Q n (%)	0 (0)	0 (0)	2 (4.3)	3 (10)	5 (16.7)	1 (0.8)	15 (16)	5 (8.2)	1 (2.3)	22 (15.4)	4 (6.5)	58 (8.3)
(n) cases at recurrence	0	1	0	3	8	14	16	8	14	33	15	112
Wild type isolates n (%)	0	1 (100)	0 (0)	3 (100)	6 (75.0)	11 (78.6)	5 (31.3)	0 (0)	4 (28.6)	1 (3.0)	0 (0)	30 (27.7)
Isolates with any SNP n (%)	0 (0)	0 (0)	0 (0)	0 (0)	2 (25.0)	3 (21.4)	11 (68.7)	8 (100)	10 (71.4)	32 (97.0)	15 (100)	81 (72.3)
Isolates with C580Y n (%)	0 (0)	0 (0)	0 (0)	0 (0)	0 (0)	0 (0)	0 (0)	0 (0)	5 (35.7)	12 (36.4)	5 (33.3)	22 (19.8)
Isolates with E252Q n (%)	0 (0)	0 (0)	0 (0)	0 (0)	0 (0)	0 (0)	7 (43.8)	2 (25.0)	0 (0.0)	4 (12.1)	1 (6.7)	14 (12.6)

*726 isolates on admission and 65 isolates from recurrence were assessed for *Pfmdr1.*
699 isolates on admission and 112 isolates from recurrence were analysed for *K13* sequence.
Both *K13* SNP and *Pfmdr1* copy number are available in 526 isolates.

Supplementary Table 3. Number of samples with each *K13* genotype by year

Mutation	2003	2004	2005	2006	2007	2008	2009	2010	2011	2012	2013	Total	
**R239Q**	0	0	0	0	0	0	0	2	0	0	0	2	Stem
**E252Q**	0	0	2	3	5	1	15	5	1	22	4	58	Stem
**D281V**	0	0	0	0	0	1	0	0	0	0	0	1	Stem
**K438N**	0	0	1	0	0	0	6	4	0	0	0	11	Propeller
**P441L**	0	0	0	0	0	1	1	1	4	7	3	17	Propeller
**F446I**	0	0	0	0	0	0	0	0	1	0	0	1	Propeller
**G449A**	0	0	0	0	0	0	0	0	0	0	1	1	Propeller
**N458Y**	0	0	1	0	0	3	0	5	4	8	6	27	Propeller
**M476I**	0	0	0	1	0	0	3	3	0	1	2	10	Propeller
**K479I**	0	0	0	0	0	0	0	0	0	0	4	4	Propeller
**A481V**	0	0	0	0	0	0	1	0	0	0	0	1	Propeller
**Y511H**	0	0	0	0	0	0	0	0	0	1	0	1	Propeller
**P527H**	0	0	0	0	0	4	0	2	0	0	0	6	Propeller
**R528G**	0	1	0	0	0	0	0	0	0	0	0	1	Propeller
**G533A**	0	0	0	0	0	0	0	0	0	2	0	2	Propeller
**N537I**	0	0	0	0	1	1	0	0	0	3	0	5	Propeller
**G538V**	0	0	2	1	0	0	4	0	4	10	3	24	Propeller
**P553L**	0	0	1	0	0	0	0	0	0	0	0	1	Propeller
**P553P**	0	1	0	0	0	0	0	0	0	0	0	1	Propeller
**R561H**	1	0	0	0	0	1	10	3	1	15	2	33	Propeller
**P574L**	0	0	0	0	0	1	1	2	0	0	4	8	Propeller
**C580Y**	0	0	0	1	0	1	0	3	10	38	20	73	Propeller
**P667Q**	0	0	0	0	0	0	0	0	0	1	0	1	Propeller
**P667T**	0	0	0	0	1	0	0	2	0	0	0	3	Propeller
**A675V**	0	0	0	0	1	1	3	5	2	8	3	23	Propeller
**WT**	14	47	40	24	22	109	50	24	17	27	10	384	
**Total**	15	49	47	30	30	124	94	61	44	143	62	699	

Supplementary Tables
